# The PRAISE study: A prospective, multi-center, randomized, double blinded, placebo-controlled study for the evaluation of iloprost in the early postoperative period after liver transplantation (ISRCTN12622749)

**DOI:** 10.1186/1471-2482-13-1

**Published:** 2013-01-29

**Authors:** Erik Bärthel, Falk Rauchfuß, Heike Hoyer, Maria Breternitz, Karin Jandt, Utz Settmacher

**Affiliations:** 1 Department of General, Visceral and Vascular Surgery, Jena University Hospital, Erlanger Allee 101, D-07740, Jena, Germany; 2 Institute of Medical Statistics, Information Sciences and Documentation, Jena, Germany; 3 Center for Clinical Studies, Jena, Germany; 4 Center for Sepsis Control and Care, Jena University Hospital, Friedrich Schiller University, Jena, Germany

**Keywords:** Liver transplantation, Primary graft dysfunction, Initial non-function, Ischemia-reperfusion injury, Prostaglandins

## Abstract

**Background:**

Liver graft dysfunction can deteriorate to complete organ failure and increases perioperative morbidity and mortality after liver transplantation. Therapeutic strategies reducing the rate of graft dysfunction are of current clinical relevance. One approach is the systemic application of prostaglandins, which were demonstrated to be beneficial in reducing ischemia-reperfusion injury. Preliminary data indicate a positive effect of prostacyclin analogue iloprost on allograft viability after liver transplantation. The objective of the study is to evaluate the impact of iloprost in a multi-center trial.

**Methods/Design:**

A prospective, double-blinded, randomized, placebo-controlled multicenter study in a total of 365 liver transplant recipients was designed to assess the effect of intravenous iloprost after liver transplantation. Primary endpoint will be the primary graft dysfunction characterized as presentation of one or more of the following criteria: ALAT or ASAT level > 2000 IU/ml within the first 7 postoperative days, bilirubine ≥ 10 mg/dl on postoperative day 7; INR ≥ 1.6 on postoperative day 7 or initial non-function. Secondary endpoints are parameters of post-transplant morbidity, like rates of infections, biliary complications, need of clotting factors or renal replacement therapy and the graft and patient survival.

**Discussion:**

A well-established treatment concept to avoid graft dysfunction after liver transplantation does not exist at the moment. If the data of this research project confirm prior findings, iloprost would improve the general outcome after liver transplantation.

**Trial Registration:**

German Clinical Trials Register: DRKS00003514. Current Controlled Trials Register: ISRCTN12622749.

## Background

The incidence of primary graft dysfunction (PDF) after LT is approximately 25% within a range of 9.3 to 43.7% based on different definitions characterizing PDF [1-5]. Initial non-function (INF) develops in up to 6% of all considered cases and requires urgent retransplantation [6].

Risk factors are prolonged cold ischemia time or higher degree of graft steatosis and subsequent development of severe hepatic ischemia/reperfusion injury (IRI) [5,7]. A cascade of cellular events results in microcirculatory flow disturbance. This may be distinguished as “no-flow” indicating capillary perfusion failure on the one hand, or “reflow-paradox” including activation of the leukocyte-endothelium interaction, release of toxic mediators, and impairment of the endothelial barrier on the other hand [8,9]. There are, obviously, further different factors all contributing to an insufficient function of liver grafts and its detrimental effects on the overall outcome after LT: recipient’s condition, the donor data, the organ conservation as well as the immunosuppression and the antibiotic regime in the perioperative period. All of these factors may influence the performance of the graft with possible development of graft dysfunction or non-function as undesired outcomes.

Due to the persisting shortage of donor the transplant centers are forced to accept so called marginal organs. The more criteria of a marginal organ are met, the higher is the risk for development of graft dysfunction [10]. Therapeutic strategies reducing the rate of organ dysfunction are of current clinical relevance. One approach is the systemic application of prostaglandin derivatives. In particular, the prostaglandins E_1_ (PGE_1_) and I_2_ (PGI_2_) showed beneficial protective effects with respect to IRI by improvement of tissue perfusion and by protection of endothelial cells studied *in vitro* and *in vivo*. Both prostaglandins induce vasodilatation, inhibit platelet aggregation and activation of leukocytes and exert a variety of “cytoprotective” effects. PGI_2_ is believed to play a superior role with respect to inhibition of platelet aggregation via PGE_1_ and also to inhibit the production of inflammatory cytokines. Therefore PGE_1_ and PGI_2_ are supposed to attenuate reperfusion injury.

There is a lack of systematic studies on the effects of synthetic PGI_2_ analogue iloprost. Many authors have reported the clinical use of the iloprost over the last two decades. Based on its ability to induce vasodilatation, it has traditionally been utilized in the treatment of pulmonary hypertension [11]. Off-label use of iloprost as an adjuvant in elective abdominal aortic aneurysm surgery and after reconstructive surgery for acute lower limb ischemia has been reported [12,13]. In these studies, the PGI_2_ infusion was associated with a significant reduction of morbidity and mortality suggesting an improved systemic perfusion and reduced secondary end-organ damage.

From 2006 to 2008, we evaluated the impact of the PGI_2_ analogue iloprost in 80 liver transplanted patients in a prospective, randomized (1:1), open-label, single-center pilot study [14]. Our data indicate that intravenous administered PGI_2_ analogue iloprost (1 ng/kg BW/min) improves the graft function, particularly in the early postoperative period. We observed ten cases of PDF according to the definition of Haller et al. [15]: eight in the control group (n = 8/40, 20%), and two in the iloprost group (n = 2/40, 5%) (relative risk 0.25, 95% confidence interval (0.05, 1.11). The relative risk did not change after adjustment to three or more extended-donor criteria (EDC). Four out of 40 patients of the control group but none of the iloprost group underwent liver retransplantation due to INF (p = 0.12).

The definition of graft dysfunction after LT varies in the literature. There is currently no standardized terminology [7,10,15-17]. Besides, in the last years, there has been a shift toward abandoning the routine use of T-tubes for reconstruction of the biliary tract [18]. Therefore, in a post-hoc analysis we applied a distinct definition of PDF, which was recently validated by Olthoff et al. [19]. This definition utilizes objective post-transplant criteria that were highly associated with graft loss and patient mortality. According to Olthoff et al., we observed 27 cases of PDF (treatment group: n = 11/40 (27.5%); control group: n = 16/40 (40%)) resulting in a relative risk of 0.69 with 95% confidence interval (0.36, 1.30).

The results of our pilot study are encouraging, but due to the number of patients enrolled, the effect of iloprost on early graft function did not reach statistical significance. There is, however, preliminary evidence to suggest beneficial effects in this particular clinical scenario. The clinical outcome after LT is affected by various donor and recipient characteristics such as graft steatosis, long ischemic period or renal insufficiency prior to LT. In order to define the specific impact of PGI_2_ analogue iloprost on liver graft function, a multicenter, randomized, double-blinded clinical trial including an appropriate number of patients is justified [20]. This is the objective of our PRAISE study.

## Methods/Design

The PRAISE study is a prospective, multi-center, randomized, double blinded (patients and investigators), placebo-controlled clinical trial according to the German drug legislation for evaluation of iloprost in the early postoperative period after LT.

The investigational medicinal product is iloprost (Ilomedin®, Bayer Vital GmbH, Leverkusen, Germany). The control group receives placebo. Patients will be assigned to treatment at the ratio of 1:1 according to a computer generated randomization list by means of nQuery Advisor software. Randomization will be restricted by blocking with randomly varying block size and stratified by center. An independent statistician, who is not involved in the analyses, will prepare the randomization list. The university pharmacy of Heidelberg, Germany, will provide the trial medication to the participating centers by sequentially numbered sealed identical boxes according to the allocation sequence.

The end of trial for each patient is reached 180 days after LT. The follow-up period will be 6 months with assessments 9 and 12 months after LT.

### Study endpoints

The primary endpoint of this trial is the rate of primary graft dysfunction after liver transplantation. It will be characterized as presentation of one or more of the following criteria in accordance with Olthoff et al [19]: ALAT or ASAT level > 2000 IU/ml within the first 7 postoperative days; bilirubin ≥ 10 mg/dl on postoperative day 7; INR ≥ 1.6 on postoperative day 7 or as occurrence of initial non-function (INF) defined as graft loss, retransplantation or patient death within 14 days after initial LT not secondary to hepatic artery thrombosis (HAT), biliary complication, recurrent disease or acute rejection. Based on the results of previous investigations a relative reduction of PDF incidence of 50% by iloprost compared to placebo will be expected.

Due to the immunologic capabilities even of the liver, graft dysfunction may lead to an increased incidence of hospital acquired infections and/or sepsis. For this reason, the incidence of infectious complications up to 28 days after LT was defined as an important secondary endpoint. Further secondary endpoints are the rate of INF (described above), the graft and patient survival (28, 180 days after LT), the clotting factor substitution up to day 28 after LT, the rate of biliary complications, the requirement for liver dialysis, the postoperative renal replacement therapy (28, 180 days after LT) and the length of intensive care unit (ICU) stay and hospital stay. The change in SOFA score from day 1 to day 7 after LT is used as a surrogate secondary endpoint predictive for morbidity and mortality after LT. The levels of liver enzymes (ASAT/ALAT) may represent the severity of graft injury due to ischemia/reperfusion and the levels of Quick’s value/INR and Factor V and the ICG-PDR are important parameters of the synthetic capacity of the graft. Therefore, the course of these laboratory data until day 7 after LT is also fixed as secondary endpoint. Occurrence of bleeding complications, circulatory instability and pulmonary disturbance during trial intervention as well as incidence of adverse events will be assessed for patient safety. During the post-trial follow-up graft and patient survival and biliary complications will be additionally evaluated 9 and 12 months after LT.

Further objectives will be explored within the scope of a scientific supporting program. Liver biopsies, taken during cold ischemia, after reperfusion and seven days after LT will be performed for histopathological and pangenomic gene expression studies. Furthermore, blood samples will be collected for “multiplex”-analysis of cytokines, chemokines and growth factors in order to investigate a possible modulating effect of iloprost on the inflammatory response after LT.

Infection is one of the leading causes of morbidity and mortality after LT. More than 50% of liver transplant recipients have infections in the first year after transplantation, whereas the majority of bacterial infections occur within 2 months after transplantation [21]. As the differentiation between infectious and non-infectious etiology of the so called SIRS (systemic inflammatory response syndrome) often requires complex and long-lasting tests valuable time is often lost until the definitive diagnosis of sepsis can be made. There is a new approach (SIQnature®, SIRS-Lab GmbH, Jena, Germany) that allows an analysis within one working day and facilitates the clinical interpretation according to a score. The data have not yet been established in patients receiving immunosuppression. Therefore, within the study setting blood samples will be taken for the evaluation of the SIQnature®-test.

### Study population and setting

Female and male patients aged 18 years and above who are receiving a full-size liver graft are eligible for the trial. Complete inclusion and exclusion criteria are displayed in Table 1. According to the experience from the pilot study [14] about 83% of primarily enrolled patients reached respiratory and circulatory stable conditions after LT as precondition for trial medication. To randomize 356 patients after LT written informed consent has to be obtained from about 430 patients fulfilling the study criteria observable before LT. In case of patients not able to give informed consent by themselves informed consent has to be given by the legal representative. Patients will be recruited in several German transplant centers. Recruitment was started in April 2012.

**Table 1:** Criteria for inclusion and exclusion of patients

**Inclusion criteria**	**Exclusion criteria**
Full-size liver transplantation	Split liver transplantation or living donor related liver transplantation
Informed consent of the patient or legal representative	Participation on other clinical trials 30 days prior to randomization
Age ≥ 18 years	Retransplantation or multivisceral transplantation
	Respiratory and/or circulatory instability (noradrenaline > 1μg/kgBW/min and FiO_2_ > 0,6)
	Conditions in which bleeding complications may be expected from the effect of Iloprost on platelets
	Known allergy or intolerance against trial medication, tacrolimus, mycophenolat mofetil, basiliximab or corticosteroids
	Severe coronary artery disease or unstable angina pectoris
	Myocardial infarction within the past 6 months prior to randomization
	Acute or chronic heart failure (NYHA II-IV)
	Cardiac arrhythmias relevant for the prognosis
	Suspected pulmonary artery congestion
	Women of child-bearing potential except women with the following criteria:
	○ post menopausal
	○ sterilization 86 weeks after bilateral ovarectomy
	○ using an effective method of contraception during the trial
	○ sexual abstinence or vasectomised partner
	Pregnancy/lactation

### Intervention

The investigational medicinal product is iloprost (Ilomedin®, Bayer Vital GmbH, Leverkusen, Germany). The control group receives placebo administered in the same dosage and application form. It is the iloprost 10 μg/ml carrier substance (ethanol, tromethamin, sodium chloride, hydrochloric acid, water for injections). Patients will be allocated to the trial medication immediately after entrance into ICU after LT if respiratory and circulatory stability is reached (noradrenaline < 1 μg/kgBW/min and FiO_2_ < 0,6). The application of the trial medication must start at the latest within the first three hours after graft reperfusion.

The trial medication will be administered continuously over a period of seven days intravenously with an infusion pump. The dosage will be body weight adapted (1 ng/kgBW/min).

During LT a marked decrease in systemic blood pressure following liver reperfusion is frequently observed [22]. This could be aggravated by iloprost due to its vasodilatatory effect. In our pilot study, clear hemodynamic alterations under iloprost application were not observed. But we recorded a higher number of pulmonary complications i.e. pleural effusion or respiratory impairment. This might be explained by a PGI_2_ induced increased intrapulmonary shunt fraction [23]. Therefore, the trial medication should be reduced in patients, who present with bleeding complications, respiratory disturbances and/or appearance of hypotension episodes.

The immunosuppressive treatment will be standardized for both groups to minimize the effects of different immunosuppressive treatment within this trial. The trial-related immunosuppression is based on retard release Tacrolimus (Advagraf®) in combination with Basiliximab (Simulect®) and Mycophenolate Mofetil. Corticosteroids might be given only as perioperative intravenously bolus.

### Study schedule

**Figure 1:**
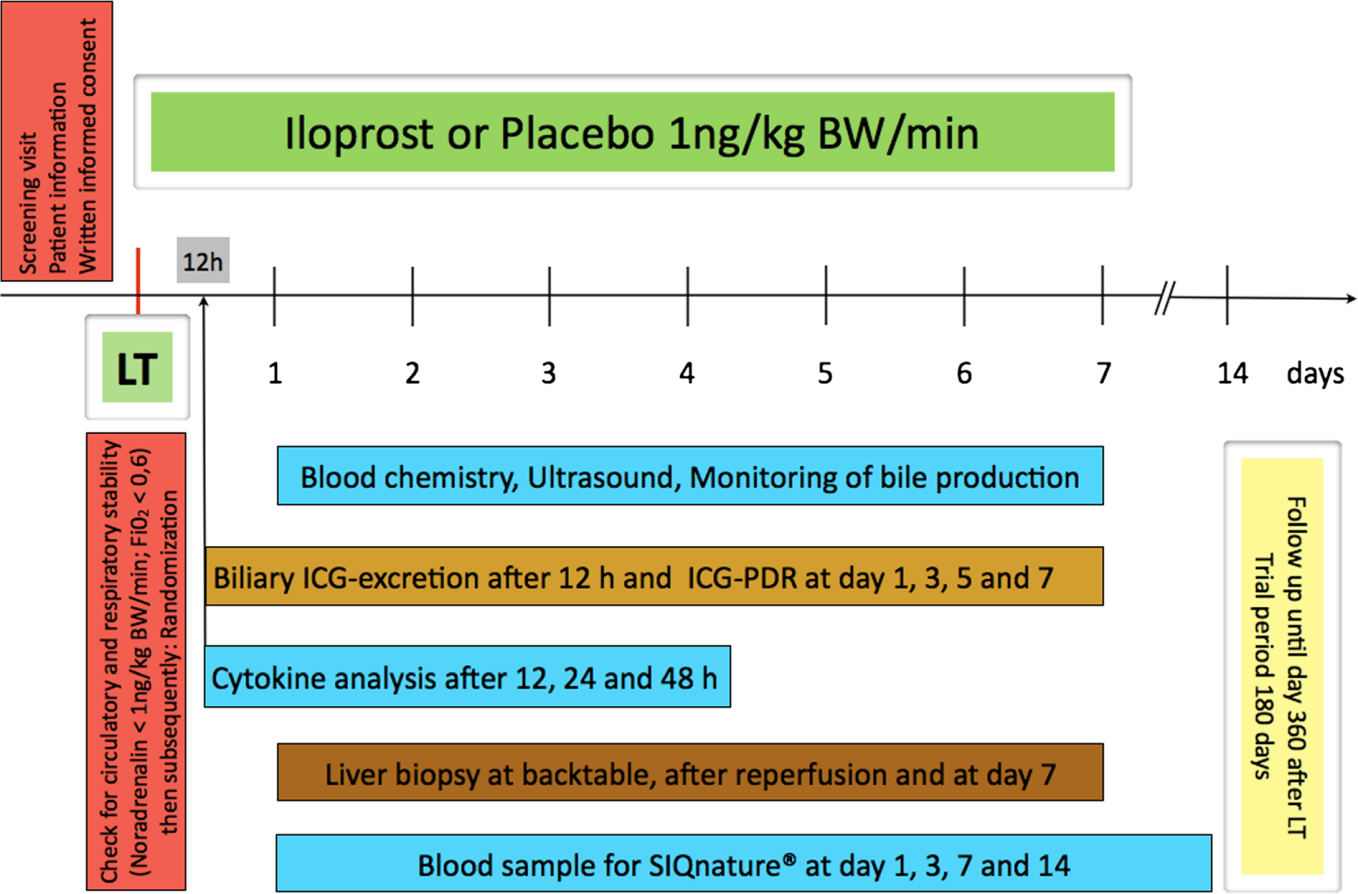
Treatment schedule.

A schematic view illustrates the Figure 1. All patients or their legal representatives must give written informed consent to participate. The screening visit is defined as assessments made after definition of need for transplantation to confirm eligibility of a particular patient for entry into this trial. During the screening the following assessments will be performed: Patient information, written informed consent, demographic data of the recipient, check for inclusion and exclusion criteria, especially for existing cardiac and pulmonary diseases.

The baseline visit starts after acceptance of the donor organ. During the baseline the following assessments will be made: concomitant medication, non-drug therapies (renal replacement therapy or liver dialysis), the current baseline data of the recipient (height, weight, lab-MELD etc.), donor criteria (age, height, weight, BMI, cause of death, virology data etc. - all data can be obtained from the Eurotransplant donor protocol) and the key laboratory data of the recipient.

During the backtable preparation, a wedge biopsy of the donor liver will be performed. After reperfusion of the graft (after finishing the bile duct anastomosis), a further liver biopsy will be done. After entrance to the ICU, a checking for circulatory and respiratory stability (noradrenaline < 1 μg/kgBW/min and FiO_2_ < 0,6) with assessment of catecholamines, mean arterial pressure, heart rate, pulmonary disturbances (blood-gas analysis, FiO_2_, PEEP, arterial oxygen saturation, pO_2_, pCO_2_) is necessary before randomization of eligible patients. After that, the trial medication will be started. For patients not fulfilling the post-LT randomization criteria of circulatory and respiratory stability, the trial is terminated.

In the treatment period, the patients receive the trial medication continuously for seven days. During this period, the following procedures should be performed daily: check for circulatory and respiratory stability, clotting factor substitution (FFP, PPSB or other coagulation factor substrates), infections, appearance of adverse events, concomitant medication, non-drug therapies, liver dialysis, renal replacement therapy, number of applicated packed red cells, the blood chemistry and ultrasound of the graft to assess quality of liver parenchyma and perfusion. In cases of an inserted T-tube (or similar) the daily bile production is monitored until the tube is clamped for the first time or at maximum up to day 7 after randomization. An optional measurement of the ICG-PDR with the LiMON-Technology (PULSION Medical Systems AG, Munich, Germany) will be performed.

The blood samples for the cytokine analysis are taken exactly 12 hours after reperfusion, at day 1 and at day 2 after randomization. Further blood samples for the SIQnature® – Test are taken on day 1, 3, 7 and 14 after randomization.

An ultrasound guided percutaneous liver biopsy is performed seven days after LT. In reasonable single cases the percutaneous liver biopsy may be abandoned if the biopsy leads to excessive endangerment of the patient or the graft.

Adverse events (AE) occurring after randomization will be assessed during the trial period up to day 180 after LT. Serious AEs will be managed according to the requirements of the German drug legislation.

### Sample size calculation

The hypotheses to be proved for PDF as primary endpoint at a two-sided 0.05 significance level are:

H0: Incidence (PDF) _Iloprost_ = Incidence (PDF) _Placebo_

HA: Incidence (PDF) _Iloprost_ ≠ Incidence (PDF) _Placebo_

The PDF definition used for this trial was introduced by Olthoff et al. [19] and validated in a population of 297 liver transplant recipients in the United States who underwent transplantation in 2004-2005 at three centers. PDF incidence was 23.2%. We hypothesize that PDF incidence can be reduced by 50% in patients treated with iloprost compared to placebo (relative risk RR = 0.5). If, according to Olthoff, an incidence of 23% will be used as baseline incidence for the placebo group a two-sided Chi^2^-test will have 80% power to detect the clinically relevant relative risk reduction of 50% when the sample size in each group is 169 (nQuery Advisor 6.01). If the baseline incidence of PDF will be 40% as observed in the pilot study on the basis of the Olthoff definition the power will be 98% to detect the postulated effect of RR = 0.5 for the same sample size of 169 in each group. Taking into account a post-randomization dropout rate for primary endpoint data of 5% a total of 356 patients should be randomized.

### Statistical analysis

Data will be analyzed for the Clinical Study Report to EC and the regulatory authority when data from the first 180 days post-LT from all patients are declared complete and accurate (end of trial). A follow-up analysis will be performed after all patients complete the follow-up period of 12 months post-LT.

The intention-to-treat (ITT) population as the primary analysis data set includes all randomized patients receiving at least one dose of trial medication. Primary and secondary efficacy data will be analyzed by randomized treatment assignment. The per-protocol (PP) population includes all randomized patients who are most compliant with the protocol. Included patients receive at least 80% of planned trial medication. They complete the study without major protocol deviations. Major protocol deviations will be identified and assignment to PP population will be done at the blind review prior to the 180-day data base lock. The safety analysis population will include all patients who receive at least one dose of trial medication. Safety data will be analyzed by the treatment assignment actually received.

For primary endpoint, the treatment effect will be estimated as relative risk and odds ratio with 95% confidence interval (CI) and will be tested by Chi^2^-test. Analysis of PDF will be possibly adjusted for centre by logistic regression with random effects. To evaluate the robustness of results the analysis is repeated for the PP-population. In explorative analyses the treatment effect will be further adjusted to known prognostic donor criteria (donor age and BMI, donor serum peak sodium, cold and warm ischemia time) within a logistic regression model possibly with random center effects.

The secondary endpoint analyses will be performed in the ITT population. Incidence of events by treatment group and relative risks with 95% CI will be estimated for occurrence of any infection, initial non-function, clotting factor-substitution, renal replacement therapy and biliary complications. The Chi^2^-test will be used for statistical testing. Length of ICU and hospital stay will be described by quartiles, means and SD. Hodge-Lehmann estimates with 95% CI will be calculated as measure of mean differences, Mann-Whitney U-test is used for significance testing. Rates of graft or patient survival will be estimated by the Kaplan-Meier method at pre-specified time points. Kaplan-Meier curves will be compared by the log-rank test. The hazard ratio with 95% CI is estimated by the Cox regression model assuming proportional hazards. The course of laboratory data over time will be analyzed by linear mixed models using raw or transformed data. Change in SOFA-score will be analyzed by two-sample t-test. Patients with missing secondary endpoint data will be excluded from the respective analysis. There is no interim analysis planned for efficacy. Annual safety reports will be generated for the German Competent Authority and the Data Management and Safety Board.

### Ethical and legal aspects

Sponsor of this investigator-initiated trial is the Friedrich Schiller University Jena. The procedures set out in the trial protocol are designed to ensure that all persons involved in the trial abide by ICH-GCP guideline, the ethical principles described in the applicable version of the Declaration of Helsinki and the German drug legislation. The trial was approved by the ethics committee of the Friedrich Schiller University Jena, Faculty of Medicine and the German Competent Authority (BfArM) (EudraCT-Number: 2010-022660-12). The study is registered at the German Clinical Trials Register (DRKS00003514) and in the Current Controlled Trial Register (ISRCTN12622749).

### Responsibilities

Coordinating investigator: Prof. Dr. Utz Settmacher, Head of the Department of General, Visceral and Vascular Surgery, Jena University Hospital, Germany.

Trial statistician: Dr. Heike Hoyer, Institute of Medical Statistics, Information Sciences and Documentation, Jena University Hospital, Germany.

Project Management: Department of General, Visceral and Vascular Surgery, Center for Clinical Studies, Jena University Hospital, Germany.

Clinical monitoring, safety and data management: Coordination Centre for Clinical Trials (KKS), University Halle, Germany.

## Discussion

Due to the shortage of donor organs, transplant centers are forced to accept so-called marginal organs. Thus, there is a significant interest in the development of new strategies to prevent graft dysfunction and early graft loss. Apart from technical complications (vascular and biliary), acute or chronic rejection, PDF accounts for the majority of indications for re-transplantation [24]. The cause of graft dysfunction is multi-factorial, but IRI plays an important role as being characterized by a severe impairment of organ microcirculation [25]. In several experimental models, prostaglandins (PG) protected the liver from severe damage induced by ischemia and reperfusion [26-29]. Several authors reported a significant decrease of liver enzyme levels after application of PG and a favorable clinical course [30,31]. Nevertheless, its prophylactic administration after LT is still a subject of discussion, because the investigators did not identify a beneficial effect on graft function itself [32,33].

The aim of our study is the investigation of the impact of continuous intravenous administration of PGI_2_ analogue iloprost after LT in order to prevent graft dysfunction.

In order to define the specific impact of PGI_2_ analogue iloprost on liver graft function, this multi-center study including a larger number of patients and will enable reliable risk stratification according to the MELD score or extended donor criteria. This upcoming trial will be under the patronage of the German Transplantation Society.

## Abbreviations

AE: Adverse event; ALAT: Alanin-amino-transferase; ASAT: Aspartat-amino-transferase; BMI: Body mass index; BW: Body weight; EC: Ethics committee; FFP: Fresh frozen plasma; FiO_2_: Fraction of inspiratory oxygen; GCP: Good clinical practice; HAT: Hepatic artery thrombosis; ICG: Indocyanine green; ICU: Intensive care unit; INF: Initial non-function; INR: International normalized ratio; IRI: Ischemia/reperfusion injury; ITT: Intention-to-treat; lab-MELD: Laboratory based model for end-stage liver disease; LT: Liver transplantation; pCO_2_: Carbon dioxide partial pressure; PDF: Primary graft dysfunction; PDR: Plasma disappearance rate; PEEP: Positive endexpiratory pressure; PG: Prostaglandin/-s; pO_2_: Oxygene partial pressure; RR: Relative risk; SIRS: Systemic inflammatory response syndrome; SOFA: Sequential organ failure assessment.

## Competing interests

EB received payment for a conference lecture and travel grants from Astellas Pharma GmbH, Munich, Germany. FR received payment for a conference lecture from BAYER Vital GmbH, Leverkusen, Germany. FR and US received travel grants from Astellas Pharma GmbH, Munich, Germany. None of the authors holds any stocks or shares in the funding organizations. There are no patent issues. All authors declare that they have no financial or non-financial competing interests.

## Authors’ contributions

All authors contributed to the accomplishment of this manuscript. EB wrote the manuscript. EB, FR, MB and HH made substantial contributions to conception and design of the study protocol. EB, FR, MB, KJ and HH wrote the study protocol and revised the manuscript. HH provided the statistical design of the study. US participated in the design and coordination of the trial. All authors read and approved the manuscript.

## Funding

Funding for this study is provided by the German Ministry of Education and Research (BMBF, FKZ:01E01002), the Astellas Pharma GmbH, Munich, Germany and the BAYER Vital GmbH, Leverkusen, Germany. They all have no role in the design of the trial and they are not in any way involved in collecting and analyzing data or interpreting of the trial results.

## Pre-publication history

The pre-publication history for this paper can be accessed here:

http://www.biomedcentral.com/1471-2482/13/1/prepub
